# Microbiological and Histological Analysis for the Evaluation of Farmed Mussels (*Mytilus galloprovincialis*) Health Status, in Coastal Areas of Italy

**DOI:** 10.3390/pathogens9050395

**Published:** 2020-05-21

**Authors:** Roberta Battistini, Katia Varello, Valeria Listorti, Michela Zambon, Giuseppe Arcangeli, Elena Bozzetta, Danila Raffaella Francese, Carlo Ercolini, Laura Serracca

**Affiliations:** 1 Department of La Spezia, Istituto Zooprofilattico Sperimentale del Piemonte, Liguria e Valle d’Aosta, 19100 La Spezia, Italy; valeria.listorti@izsto.it (V.L.); carlo.ercolini@izsto.it (C.E.); laura.serracca@izsto.it (L.S.); 2 Department of Histopathology, Istituto Zooprofilattico Sperimentale del Piemonte, Liguria e Valle d’Aosta, 10154 Torino, Italy; katia.varello@izsto.it (K.V.); elena.bozzetta@izsto.it (E.B.); DanilaRaffaella.francese@izsto.it (D.R.F.); 3 National Reference Centre for Fish, Mollusc and Crustacean Diseases, Istituto Zooprofilattico Sperimentale delle Venezie, 35020 Legnaro (PD), Italy; mzambon@izsvenezie.it (M.Z.); garcangeli@izsvenezie.it (G.A.)

**Keywords:** shellfish, histology, *Vibrio*, Ostreid Herpesvirus-1, mussel parasites

## Abstract

Shellfish farming is a relevant economic activity in Italy. The Gulf of La Spezia is one of the major production areas for mussels: the area is characterized by the presence of numerous human activities that could harm the quality of seawater. Additionally, the presence of potentially pathogenic microorganisms may influence the health status of animals, which must be constantly monitored. To have a clear view of the health conditions of the mussels (*Mytilus galloprovincialis*) farmed in this area, microbiological, parasitological, and histological analyses were performed. The study was conducted from November 2016 to October 2017. Overall, despite the presence of potentially pathogenic microorganisms for mussels, abnormal mortality rates were not reported during the monitoring period and the histological examination revealed no significant lesions. Our study confirms that studying different aspects together is a useful tool for assessing the health conditions of mussels and points out the importance of adverse environmental conditions for the expression of the pathogenicity of microorganisms.

## 1. Introduction

Mussel farms are usually located in highly anthropized areas, although these guarantee a good supply of nutrients for mussels breeding, could represent a problem for the introduction of polluting agents that can weaken the immune system of the mussels. Several environmental contaminants can cause oxidative stress, being strongly pro-oxidant [1]. The presence of abiotic environmental stressors (variations of environmental parameters and presence of pollutants) and biological variables (variations of the physiological state in particular phases of the reproductive cycle, presence of potentially pathogenic microorganisms) can lead to particular criticalities depending on the seasonality or the performance of human activities in particular sites. In particular, among the pathogenic microorganisms, members of the genus *Vibrio* can cause mortality and therefore economic losses. *Vibrio* spp. are ubiquitous in marine environments and can be found in large quantities within bivalve mollusks. Generally, these microorganisms are normal components of mussel microflora without any pathological significance [2] and can be found in healthy mollusk tissues throughout the year; however, in particular conditions, they can interfere with their immune system affecting their vitality and be associated with mortality, particularly in juvenile forms and in larvae. Microorganisms of the genera *V. splendidus* clade, *V. harveyi* clade, and *V. aestuarianus* can be pathogenic for mollusks [3]. Among the pathogenic *Vibrio*, species belonging to the *V. splendidus* clade have recently been associated with forms of massive mortality in mussels [4]. Parasites could also be harmful to mussels: infection with *Marteilia refringens* is a listed of the World Organisation for Animal Health (OIE) disease [5] and this pathogen can cause physiological disorders and eventually death of the animals [6]. Finally, Ostreid herpesvirus (OsHV1) is a viral agent that is causing problems for oyster farming, causing immunosuppression in these animals, and promoting infection by bacterial agents [7]. OsHV-1 is also isolated from mussels [8] and it has been shown that experimentally infected mussels can exhibit the same histological changes found in infected oysters and can act as reservoirs for this microorganism [9]. The *Mytilus galloprovincialis* represents an important economic resource in the Gulf of La Spezia with a production of about 2000 tons per year. The Gulf is limited by a dam, and it is characterized by the presence of anthropogenic activities such as an important commercial and touristic port. Previous studies showed the presence in this area of potentially pathogenic microorganisms that in adverse environmental conditions were implicated in mortality outbreaks [10,11,12]. To continue the surveillance undertaken by these previous studies in the area and prevent economic losses to a regionally important traditional sector, we reported the results relating to the presence of the potential pathogenic microorganisms mentioned above and the health status of the farmed bivalves. this was achieved through a monitoring program that includes pathophysiological, microbiological, molecular, and parasitological analyses on mussels sampled in four sites in the Gulf of La Spezia. Moreover, to identify early risk factors that could interfere with the vitality of the bivalve mollusks the so-called “brown cells” were sought in the tissues of the mussels. These cells in mollusk tissues have been associated with environmental pollution [1]. They seem to intervene in the detoxification of pollutants, as they have brownish lysosomes in their cytoplasm associated with degrading and detoxifying processes. 

## 2. Results

### 2.1. Microbiological Analysis

**Figure 1:**
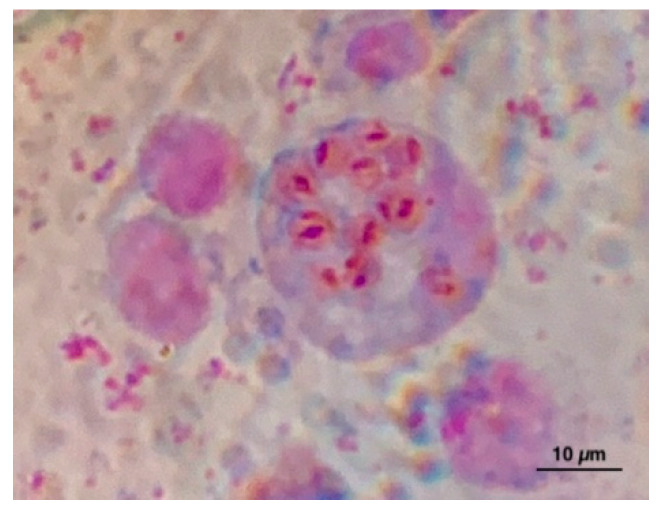
*Marteilia* spp. in a digestive gland imprint from *Mytilus galloprovincialis*.

*Vibrio splendidus* clade was detected in 50% (24/48) of shellfish sampling. For some sampling, more than one colony was isolated, overall a total of 70 strains were isolated. Virulence-related genes (*vsm* and/or *ompU*) were present in 95.8% (23/24) of the positive sampling and 68% of the isolated s trains: *vsm* positive/*ompU* negative were present in 62 strains, *vsm* negative/*ompU* positive in 1 strain, and 5 strains showed positivity for both genes. The positive samples were isolated throughout the sampling period except for July. *V. splendidus* clade was found in 9/12 months in IES, in 7/12 months in the Internal West Site (IWS), in 6/12 months in the External West Site (EWS), and only in 2/12 months in the Portovenere (PV). At all sites, the largest number of colonies was isolated in November and December. Virulence-related genes, *vsm,* were found in 80.9% (17/21) of colonies isolated by IES, in 100% (16/16) of colonies by IWS, of 95% (19/20) of colonies by EWS and in 76.9% (10/13) of colonies by PV. *OmpU* was found only in one colony by IWS. Both virulence-related genes were found together in two colonies isolated from IES and three from PV. All samples resulted in negative for *V. aestaurianus. V. harveyi* clade was detected in 12.5% (6/48) of sampling in the four sites starting from July, in total 26 strains were isolated (Table 1). In total 64/70 colonies isolated and characterized as *V. splendidus* clade by biomolecular analyses and 13/26 colonies of *V. harveyi* clade were analyzed using the MALDI-TOF (Table 2 and Table 3). Among the 64 colonies analyzed, 61 gave a result between 2300 and 3000, corresponding to a high probability of genus and species identification. In particular, 32 colonies have been identified as *V. pomeroyi*, 20 as *V. atlanticus*, 2 as *V. splendidus*, 2 as *V. celticus,* and 5 as *V. chagasii*. Three colonies gave a result between 2000 and 2299, corresponding to a certain genus identification and probably species identification. In particular, one colony was identified as *V. atlanticus*, one as *V. pomeroyi,* and one as *V. tasmaniensis*. Among the colonies of *V. harveyi* clade analyzed, five gave a result >2300 and three gave a result between 2000 and 2299 for *V. harveyi*, three were identified as *V. campbellii*, one as *V. tubiashii,* and one was not identifiable. The DNA of OsHV-1 was found in 16.6% (8/48) of samples analyzed during December, February, March, April, and May from IWS and PV sites. *Marteilia* spp. was found using the cytological method (Figure 1) in 0.67% (1/150) of samples collected in October from IWS, IES, and of samples collected in June from EWS (Table 1). Following the interpretation of the results of the OIE manual [6], the positivity found is strongly indicative of infection with *M. refringens,* without further confirmation, since the circulation of this pathogen in *M. galloprovincialis* in this area it has been known for a long time.

### 2.2. Histological Evaluations

#### 2.2.1. Gonadal Stages and Sex Determination

Of the 240 samples analyzed, 84 males and 76 females were detected. For the remaining 80 animals, sex could not be determined because they belonged to the gonadal stages I and V. The most representative gonadic stage was stage III for both sexes. In particular, the males belonging to stage II were 26, 48 to stage III, and 10 to stage IV. The females belonging to stage II were 18, 50 to stage III, and 12 to stage IV. 

#### 2.2.2. Histopathological Features

**Figure 2:**
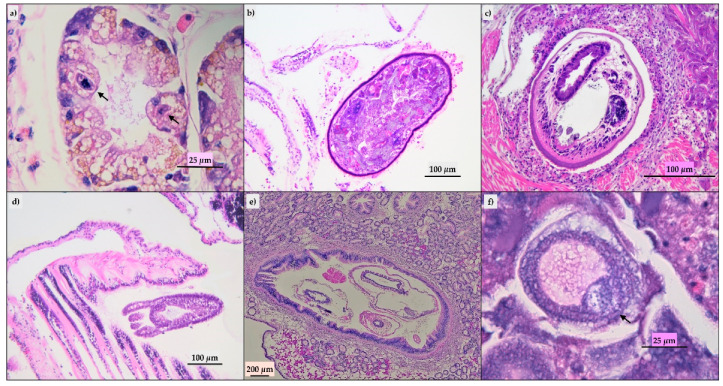
Parasites detected at histological evaluation. (**a**) Digestive gland: ciliates in the gland tubules (arrow), hematoxylin-eosin (HE); (**b**) gill: Turbellaria, HE; (**c**) adductor muscle: Trematode, HE; (**d**) gill: *Eugymnanthea inquiline*, HE.; (**e**): intestine: *Mytilicola intestinalis* in lumen, HE; (**f**) ovarium: *Steinhausia mytilovum* in oocyte (arrow), HE.

**Figure 3:**
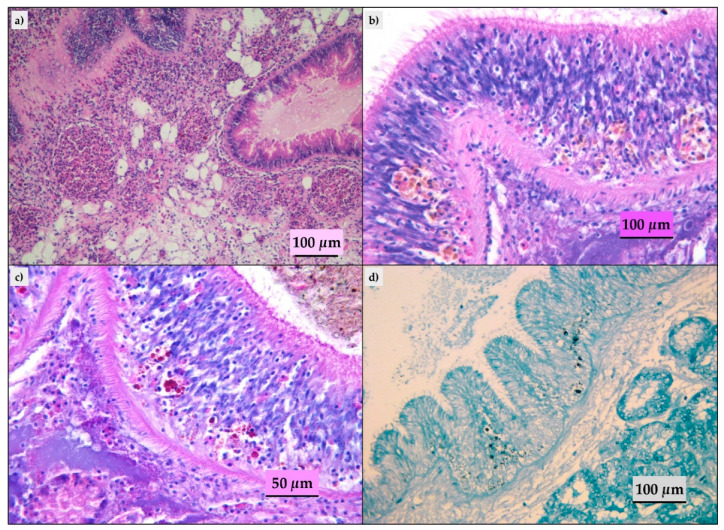
Histopathological findings in mussels analyzed. (**a**) Digestive gland: granulocytomas, HE; (**b**) intestine: lipofuscins and rare brown cells in the epithelium, HE; (**c**) intestine: lipofuscins and rare brown cells in the epithelium, Periodic Acid Schiff (PAS) stain; (**d**) intestine: lipofuscins and rare brown cells in the epithelium, Schmorl’s stain.

Ninety-three of the evaluated mussels did not show histological lesions and in the remaining animals, we detected parasitic infestation, inflammatory lesions, lipofuscins, and sporadic presence of brown cells. The parasitic infestation results were mild to moderate; ciliates (Figure 2a) and turbellaria (Figure 2b) were the parasites most frequently observed. Trematodes (Figure 2c), hydrozoa (*Eugymnanthea inquilina*) (Figure 2d), and copepod (*Mytilicola intestinalis*) (Figure 2e) were also detected, and only in one animal, the Microsporidium, *Steinhausia mytilovum*, was present in the oocytes (Figure 2f). Frequently, different parasites have been detected in the same animal. Lipofuscins and sporadic brown cells (Figure 3b) were revealed in the gastric or intestinal epithelium and in some animals related to parasitic infestation. Twenty-eight animals showed mild to moderate inflammatory lesions represented by hemocytic infiltration or granulocytomas (Figure 3a). The Periodic Acid Schiff (PAS) and Schmorl’s (Figure 3c,d) stains confirmed the presence of the lipofuscins and brown cells in the 33 animals with histological features. 

## 3. Discussion

The microbiological investigation indicated the presence of several potentially dangerous microorganisms, although no abnormal mortality was observed during the monitoring period. In particular, strains belonging to the polyphyletic group *V. splendidus* have been isolated. These strains were positive for the investigated virulence-related genes and five of them showed positivity for both genes. Microorganisms belonging to *V. splendidus* clade are often reported as normal commensals of marine animals, and it has been hypothesized that there is no correlation between phenotypic and genotypic virulence patterns [13]; however, the presence of both the investigated virulence-related genes may cause mortality, and the pathogenicity of the *Vibrio* strains can be exacerbated in mussels with a weak immune system due for instance by the occurrence of adverse environmental conditions [14,15,16]. During the monitoring period, no particular criticism in the environmental conditions have been registered, the values did not show significant deviations from the seasonal averages of the area (data not shown). MALDI-TOF analysis allowed us to identify, in *Vibrio splendidus* clade, strains considered potentially pathogenic for numerous vertebrates and marine invertebrates such as: *V. crassostreae, V. splendidus, V. tasmaniensis,* and *V. harveyi* [3]. It is interesting the identification of *V. celticus*, a pathogenic strain for clams that was previously reported between Spain and the English Channel [17]. Despite the absence of mortality events, our results highlight the presence of Ostreid Herpesvirus type 1 in mussels. The OsHV-1 is an enveloped virus and is not able to persist for a long time in the environment. It is conceivable that the mussels were positive for the presence of the virus in the host cells. In a previous study [18], the presence of viral DNA was reported in healthy oysters suggesting the existence of healthy carriers. The presence of OsHV-1 in mussels bred in the Gulf of La Spezia can represent a risk factor for the breeding of pacific oysters (*Crassostrea gigas*) and flat oysters (*Ostrea edulis*) which are bred in the same areas and for which it is well known the pathogenic potential of OsHV-1 [19], and also for the mussels themselves, although the pathogenic potential of OsHV-1 in these animals remains to be investigated. The herpesviruses can be latent in sensitive and reservoir hosts and can be reactivated depending on the host age, its physiological conditions, environmental factors, and the concomitant presence of dangerous microorganisms, thus triggering the replication and transmission of the virus [20,21].

In the current research, the prevalence of *Marteilia* spp. in mussels detected using the cytological method was very low (values less than 1%). In the past, *Marteilia refringens* was detected in *M. galloprovincialis* in the Gulf of La Spezia with a prevalence of 9.07% in 2010, 6.9% in 2011, 2.2% in 2012, and 1.17% in 2013 respectively [12]. Our data confirmed the constant circulation of the parasite in this area, although it is decreasing compared to previous years. Marteiliosis is a disease listed in the OIE and the farmed area studied has considered endemic since the parasite was first found.

Mussels rarely have negative consequences of marteiliosis, populations from different areas, with different genetic characteristics, shows different sensitivity to *Marteilia* spp. [22]; however, there could be high mortality rates related to the presence of the parasite, with important economic losses [23,24]. Although there is a low prevalence of *Marteilia* spp. in the areas examined, in stressful conditions, such as temperature rise, pollution, changes in water parameters, the prevalence could increase causing massive infestations and an increase of mortality rates in mussels. This has already happened in this area in the past, in August 2003 [12] and in February 2015 [11]. During this period in particular, the dredging operations of the dam were carried out, which probably caused changes in the conditions of the biological cycle of the parasite. Recently in France, the presence of the parasite, associated with stressful conditions due to the spawning season caused a high mortality outbreak [25]. We can say that currently, the presence of the parasite is not a problem in the farms examined; however, it is a parameter that needs to be continuously monitored to prevent economic losses. Histologically, we can assert that the exanimated mussels were in good health status. In fact, a great number of animals evaluated did not show lesions and the remaining presented mild to moderate parasitic infestation or mild focal to multifocal inflammation. Moreover, the brown cells were detected only in few mussels in which they were occasionally present in the gastric and intestinal epithelium. The presence of parasites was the most common histological finding. The parasites detected were located mainly in gills and intestine and were identified among those most frequently reported in *Mytilus galloprovincialis* [26,27,28,29,30]. The protozoa and metazoan detected are usually considered as commensals, that normally do not cause tissue damage in the animal or specific response from the host. They can cause malfunction of the infested organ only if present in large numbers. The microsporidium *Steinhausia mytilovum*, that infests the oocytes, can instead destroy the oocytes but in this case, it was present only in one animal. No massive infestations were detected in the animals analyzed, and in addition, the parasites were rarely associated with a pathologic tissue reaction. Mild inflammatory lesions (hemocytes infiltrate and granulocytomas) were mainly observed in the interstitial tissue of the digestive gland. Regarding the brown cells, defined as cells containing vesicles identified as lysosomes, in the analyzed samples they were found to be rare and, when present, in a very limited number. Their presence in the tissues, and above all their increase, indicates possible environmental pollution [31,32]. Indeed, they have been reported in the tissues of mollusks living in more polluted environments and their lipofuscins accumulation can be considered an indicator of exposure [1]. Moreover, the brown cells have also been referred by some authors as a defense mechanism against parasites [33]. In the past, in the same area, two previous studies were conducted to investigate the presence of pathogenic microorganisms and the health status of the mussels. In the case of the abnormal mortality in the mussels (50%–90%) in February 2015 microorganisms belonging to the *V. splendidus* clade were isolated. In that period there was, as previously said, a dredging process of the dam and an increase of the passage of draft ships. High turbidity of water and the presence of mud was observed in the breeding areas. During that period, a certain linking factor between the mass mortality and the ship traffic and dredging has not been found, but it is conceivable that the anthropogenic activities could have determined changes in environmental conditions causing weakness of mussels [11]. The following monitoring study conducted between October 2015 and September 2016 revealed the presence of *V. splendidus* clade and *V. harveyi* clade microorganisms, parasites, and OsHV-1, without adverse environmental conditions and a general good health status of mussels, as we observed [10]. In the Gulf of La Spezia, the mussels farming is a very important economic source, so it could be very useful to have a clear view of the health status of the mussels constantly to preserve the production.

## 4. Materials and Methods 

### 4.1. Bivalve Mollusc Samples

**Figure 4:**
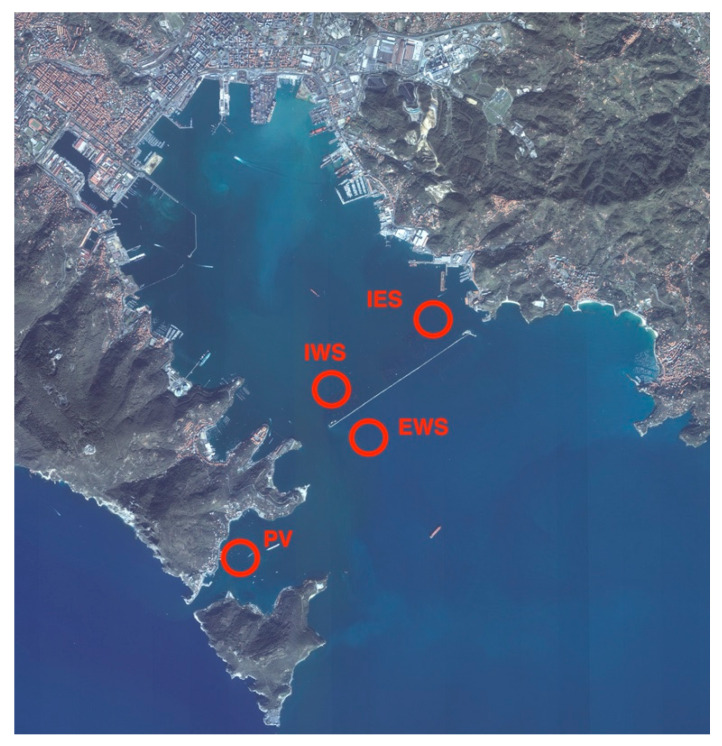
Map of the study area and sampling points location. PV: Portovenere; IWS: Internal West Site; EWS: External West Site; IES: Internal East Site. Image source: Google maps adapted by the authors.

In the period November 2016– October 2017 samples of *Mytilus galloprovincialis* were harvested monthly from four shellfish production areas in the Gulf of La Spezia (northwest of Italy). The collection sites were located two inside and two externals to the dam of the Gulf and are named: Internal West Site (IWS), Internal East Site (IES), Portovenere” (PV), and External West Site (EWS) (Figure 4). Samples were shipped refrigerated to the laboratories and analyzed for microbiological, parasitological, and histological parameters. Physico-chemical parameters of seawater (temperature, pH, salinity, turbidity, and O2) in the farming area during the period studied were within the range of annual values for the Gulf of La Spezia [34].

### 4.2. Microbiological Analyses 

#### 4.2.1. *Vibrio splendidus* clade, *V. aestuarianus*, and *V. harveyi*

For each site, a portion of mantle/gills tissues was taken from 10 mussels for a total of 10 g and added to 90 mL of Alkaline Peptone Water Salt (APWS). Then samples were diluted from 1/100 to 1/10,000 in APWS and 50 μL of each dilution was plated on Thiosulfate Citrate Bile Salts Sucrose (TCBS) plates and incubated at 20 °C for 48–96 h. Bacteria corresponding to the representative colony were sampled and total DNA was obtained by heating the colony placed in 500 µL of molecular biology grade water for 10 min at 95 °C. Total DNA thus obtained was used for the research of *V. splendidus* clade strains and *V. aestuarianus* by performing a multiplex Real-Time PCR according to IFREMER, 2013 protocol [35]. *V. harveyi* DNA was searched using a PCR assay targeting the hemolysin *hly* gene using the primer pair HLY-F and HLY-R as previously published [36]. The presence of virulence factors among colonies of *V. splendidus* clade strains isolated was assessed by a PCR targeting *vsm* and *ompU* genes [13].

#### 4.2.2. MALDI-TOF Analysis

In total, 77 strains (64 of *V. splendidus* clade and 13 of *V. harveyi* clade) were isolated as described above and analyzed using matrix-assisted laser desorption/ionization (MALDI), and mass analyzer time-of-flight (TOF). The samples were prepared according to the protocol described by Calderaro et al., 2013 [37]. The measurements were performed using the instrument Microflex LT (Maldi Biotyper, Bruker-Daltonics, Inc., Billerica, MA, USA) with the software flex Control (3.3 Bruker-Daltonics version). The instrument provides a protein profile for each bacterial strain and mass spectra between 1960 and 20132 m/z were analyzed using the MALDI Biotyper RT Classification software (version 3.1, Bruker-Daltonics) and compared with a total of 5627 reference spectra of the Bruker database and 64 Vibrionaceae reference strains supplied by National Reference laboratory for fish, crustacean, and mollusk pathology and implemented in MALDI TOF system. The resulting similarities were illustrated by a log score indicating: a high probability of genus and species identification with value >2300; a certain genus identification and probably species identification with values between 2000 and 2299; a probable genus identification with values between 1700 and 1999. For values <1699: identification is not valid. 

#### 4.2.3. Ostreid Herpesvirus-1 

DNA of Ostreid Herpesvirus-1 (OsHV-1) was searched for in mussels by taking a piece of mantle and gills. These tissues were dissected from 10 mussels for each sample and pooled. DNA was extracted from 0.025 g of each pool using a commercial kit (Cador, Qiagen) and a DNA sample was eluted in 100 µL. The molecular assay was performed by a Real-Time PCR protocol previously described [38]. 

### 4.3. Parasitological Analysis

#### *Marteilia* spp.

One hundred and fifty mussels were sampled in June and September/October from each studied area and checked for the presence of *Marteilia* spp. by cytological analysis. A little piece of the digestive gland was used for tissue imprints. The imprints were air-dried, stained with Hemacolor Kit (Merck), and observed by microscopy 1000× using a Nikon Optiphot-2 microscope [6]. 

### 4.4. Histological Analyses

Mussels (5 individuals from each sampling site) were sampled for histological evaluations, for a total of 240 animals. The whole body of each mussel was fixed for 48 h in Carson’s fixative at room temperature. A section of the mussel containing the digestive gland, gills, gut, gonads, mantle, and adductor muscle was dehydrated in a graded series of ethanol and then paraffin-embedded. Samples were cut in 4 ± 2 μm serial sections and stained with hematoxylin-eosin (HE) standard stain to evaluate the histopathological features, the presence of brown cells, and the gonadal maturation stage. Thirty-three samples, where lipofuscins and brown cells were histologically identified, have been stained with Periodic Acid Schiff stain (PAS) to detect glycogen, mucin, mucoprotein, and glycoproteins and Schmorl’s stain to detect melanin pigment, argentaffin granules, and lipofuscins. Slides were evaluated microscopically at increasing magnifications (10×, 20×, and 40×) by a light microscope (Zeiss Axio Scope A1, Germany). When parasites were present in the histological slides, they were identified based on the literature [28,29,30].

The gonadal stages were classified based on the guidelines of the Italian National Reference Laboratory for fish, crustacean, and mollusk, summarized thus:

stage I: early gametogenesis (Sex category is difficult to distinguish at this stage).
stage II: advanced gametogenesis.
stage III: mature gonad.
stage IV: spawned gonad.
stage V: resting gonad.

Identification of sex category (male and female) was assessed on the animals belonging to stages II, III, and IV because in the stages I and V the sex category is difficult to distinguish. 

## 5. Conclusions

In conclusion, no particular differences were observed throughout the different farming areas, and a good health status was found across all sampling, despite the presence of potentially pathogenic microorganisms. This evidence supports the hypothesis of the importance of environmentally stressful conditions leads to a weakening of mussels that can further the expression of pathogenicity effects of the circulating microorganisms. Continuous monitoring of the diversity, abundance, and pathogenic potential of microorganisms circulating in shellfish farmed area, as well as of environmental parameters, can be a useful tool to help quickly understand the emergence of possible epidemics and future outbreaks. Therefore, monitoring in the investigated area will also continue in the future.

## Figures and Tables

**Table 1:** Detection of microbiological parameters in mussels for date and sites. IWS: Internal West Site, IES: Internal East Site, PV: Portovenere, and EWS: External West Site.

Site	Month	*V. splendidus* Clade (*vsm* and/or *ompU*)	*V. harveyi* Clade	*V. aestuarianus*	OsHV-1	*Marteilia* spp.
**IWS**	Nov-16	1/1 (1 *vsm*)	0/0	0/1	0/1	n.d.
	Dec-16	10/10 (10 *vsm*)	0/0	0/10	0/1	n.d.
	Jan-17	1/1 (1 *vsm*)	0/0	0/1	0/1	n.d.
	Feb-17	0/0	0/0	0/0	1/1	n.d.
	Mar-17	1/1 (1 *vsm*)	0/0	0/1	1/1	n.d.
	Apr-17	1/2 (1 *vsm*)	0/1	0/2	1/1	n.d.
	May-17	0/0	0/0	0/0	0/1	n.d.
	June-17	1/2 (1 *vsm*)	0/1	0/2	0/1	0/150
	July-17	0/0	0/0	0/0	0/1	n.d.
	Aug- 17	0/7	2/7	0/7	0/1	n.d.
	Sep-17	1/1 (1 *vsm*)	0/0	0/1	0/1	n.d.
	Oct-17	0/4	0/4	0/4	0/1	1/150
	**Total**	16/29 (16 *vsm*)	2/13	0/29	3/12	1/300
**IES**	Nov-16	7/7 ((5 *vsm and* 2 (*vsm+ompU)*)	0/0	0/7	0/1	n.d.
	Dec-16	5/5 (5 *vsm*)	0/0	0/5	0/1	n.d.
	Jan-17	2/2 (1 *vsm* and 1 *ompU*)	0/0	0/2	0/1	n.d.
	Feb-17	2/2 (2 *vsm*)	0/0	0/2	0/1	n.d.
	Mar-17	1/1 (1 *vsm*)	0/0	0/1	0/1	n.d.
	Apr-17	1/1 (1 *vsm*)	0/0	0/1	0/1	n.d.
	May-17	1/1 (0)	0/0	0/1	0/1	n.d.
	June-17	0/2	0/2	0/2	0/1	0/150
	July-17	0/3	1/3	0/3	0/1	n.d.
	Aug- 17	1/4 (1 *vsm*)	0/3	0/4	0/1	n.d.
	Sep-17	0/7	4/7	0/7	0/1	n.d.
	Oct-17	1/3 (1 *vsm*)	0/2	0/3	0/1	1/150
	**Total**	21/38 (17 *vsm, 1 ompU* and 2 (vsm*+ompU*))	5/17	0/38	0/12	1/300
**EWS**	Nov-16	6/9 (5 *vsm*)	0/3	0/9	0/1	n.d.
	Dec-16	10/10 (10 *vsm*)	0/0	0/10	0/1	n.d.
	Jan-17	0/0	0/0	0/0	0/1	n.d.
	Feb-17	0/0	0/0	0/0	0/1	n.d.
	Mar-17	1/1 (1 *vsm*)	0/0	0/1	0/1	n.d.
	Apr-17	1/1 (1 *vsm*)	0/0	0/1	0/1	n.d.
	May-17	1/1 (1 *vsm*)	0/0	0/1	0/1	n.d.
	June-17	0/2	0/2	0/2	0/1	1/150
	July-17	0/0	0/0	0/0	0/1	n.d.
	Aug- 17	0/1	0/1	0/1	0/1	n.d.
	Sep-17	0/2	1/2	0/2	0/1	0/150
	Oct-17	1/7 (1 *vsm*)	3/6	0/7	0/1	n.d.
	**Total**	20/34 (19 *vsm*)	4/14	0/34	0/12	1/300
**PV**	Nov-16	3/4 (3 (*vsm+ompU*))	0/1	0/4	0/1	n.d.
	Dec-16	10/10 (10 *vsm*)	0/0	0/10	1/1	n.d.
	Jan-17	0/0	0/0	0/0	0/1	n.d.
	Feb-17	0/0	0/0	0/0	1/1	n.d.
	Mar-17	0/0	0/0	0/0	1/1	n.d.
	Apr-17	0/0	0/0	0/0	1/1	n.d.
	May-17	0/0	0/0	0/0	1/1	n.d.
	June-17	0/0	0/0	0/0	0/1	0/150
	July-17	0/0	0/0	0/0	0/1	n.d.
	Aug- 17	0/16	15/16	0/16	0/1	n.d.
	Sep-17	0/3	0/3	0/3	0/1	0/150
	Oct-17	0/1	0/1	0/1	0/1	n.d.
	**Total**	13/34 (10 *vsm and* 3 (vsm*+ompU*))	15/21	0/34	5/12	0/300

n.d., not done.

**Table 2:** *V. splendidus* clade isolates MALDI-TOF results. Legend: <1699: Identification not valid. From 1700 to 1999: Probably genus identification. From 2000 to 2299: certain genus identification and probably species identification. >2300: High probability of genus and species identification.

Num Colony	1st Species Identified	Score	2nd Species Identified	Score
1	*V. atlanticus*	2318	*V.splendidus*	2187
2	*V. splendidus*	2382	*V. atlanticus*	2373
3	*V. atlanticus*	2482	*V. splendidus*	2253
4	*V. pomeroyi*	2476	*V. gigantis*	2441
5	*V. pomeroyi*	2504	*V. celticus*	2405
6	*V. pomeroyi*	2446	*V. chagasii*	2218
7	*V. pomeroyi*	2456	*V. chagasii*	2336
8	*V. pomeroyi*	2473	*V. celticus*	2419
9	*V. atlanticus*	2511	*V. splendidus*	2505
10	*V. tasmaniensis*	2232	*V. atlanticus*	2194
11	*V. pomeroyi*	2525	*V. celticus*	2480
12	*V. pomeroyi*	2530	*V. celticus*	2389
13	*V. pomeroyi*	2503	*V. celticus*	2329
14	*V. pomeroyi*	2631	*V. celticus*	2549
15	*V. celticus*	2416	*V. pomeroyi*	2376
16	*V. pomeroyi*	2432	*V. celticus*	2411
17	*V. pomeroyi*	2613	*V. celticus*	2432
18	*V. pomeroyi*	2496	*V. celticus*	2434
19	*V. atlanticus*	2320	*V. splendidus*	2315
20	*V. chagasii*	2435	*V. chagasii*	2318
21	*V. pomeroyi*	2416	*V. gigantis*	2381
22	*V. atlanticus*	2045	*n.i.* ^1^	-
23	*V. pomeroyi*	2498	*V. celticus*	2482
24	*V. pomeroyi*	2458	*V. celticus*	2437
25	*V. atlanticus*	2456	*V. tasmaniensis*	2352
26	*V. pomeroyi*	2537	*V. celticus*	2470
27	*V. atlanticus*	2331	*V. splendidus*	2174
28	*V. pomeroyi*	2565	*V. celticus*	2433
29	*V. pomeroyi*	2513	*V. gigantis*	2508
30	*V. atlanticus*	2610	*V. splendidus*	2494
31	*V. pomeroyi*	2521	*V. celticus*	2405
32	*V. pomeroyi*	2417	*n.i.*	-
33	*V. celticus*	2429	*V. pomeroyi*	2393
34	*V. pomeroyi*	2412	*V. celticus*	2310
35	*V. splendidus*	2483	*V. atlanticus*	2415
36	*V. pomeroyi*	2438	*V. celticus*	2333
37	*V. pomeroyi*	2434	*V. celticus*	2403
38	*V. pomeroyi*	2477	*V. celticus*	2430
39	*V. pomeroyi*	2361	*V. celticus*	2324
40	*V. pomeroyi*	2269	*V. chagasii*	2088
41	*V. pomeroyi*	2461	*V. celticus*	2358
42	*V. pomeroyi*	2449	*V. celticus*	2346
43	*V. pomeroyi*	2558	*V. chagasii*	2454
44	*V. pomeroyi*	2503	*V. celticus*	2391
45	*V. pomeroyi*	2427	*V. celticus*	2355
46	*V. atlanticus*	2545	*V. splendidus*	2296
47	*V. atlanticus*	2489	*V. tasmaniensis*	2338
48	*V. atlanticus*	2598	*V. splendidus*	2570
49	*V. atlanticus*	2418	*V. tasmaniensis*	2356
50	*V. atlanticus*	2318	*V. splendidus*	2187
51	*V. atlanticus*	2482	*V. splendidus*	2254
52	*V. atlanticus*	2416	*V. splendidus*	2199
53	*V. atlanticus*	2353	*V. splendidus*	2211
54	*V. atlanticus*	2513	*V. splendidus*	2311
55	*V. atlanticus*	2462	*V. splendidus*	2352
56	*V. atlanticus*	2437	*V. splendidus*	2231
57	*V. atlanticus*	2482	*V. splendidus*	2281
58	*V. pomeroyi*	2368	*V. chagasii*	2254
59	*V. atlanticus*	2619	*V. splendidus*	2386
60	*V. chagasii*	2448	*V. pomeroyi*	2282
61	*V. chagasii*	2389	*V. celticus*	2231
62	*V. pomeroyi*	2334	*V. kanaloae*	2092
63	*V. chagasii*	2452	*V. pomeroyi*	2253
64	*V. chagasii*	2318	*V. pomeroyi*	2213

^1^ n.i.: not identifiable.

**Table 3:** *V. harveyi* clade isolates MALDI-TOF results. Legend: <1699: Identification not valid. From 1700 to 1999: Probably genus identification. From 2000 to 2299: certain genus identification and probably species identification. >2300: High probability of genus and species identification.

Num Colony	1st Species Identified	Score	2nd Species Identified	Score
1	*V. harveyi*	2218	*V. campbellii*	2211
2	*V. harveyi*	2301	n.i.^1^	-
3	*V. tubiashii*	2746	*V. orientalis*	2130
4	*V. harveyi*	2404	n.i.^1^	-
5	n.i.	-	n.i.^1^	-
6	*V. campbellii*	2264	*V. harveyi*	2256
7	*V. campbellii*	2327	*V. harveyi*	2299
8	*V. harveyi*	2264	n.i.^1^	-
9	*V. harveyi*	2301	*V. campbellii*	2288
10	*V. harveyi*	2128	n.i.^1^	-
11	*V. harveyi*	2361	*V. campbellii*	2333
12	*V. harveyi*	2301	n.i.^1^	-
13	*V. campbellii*	2339	*V. harveyi*	2287

^1^ n.i.: not identifiable.
